# Clinical Audit of Physical Examinations and Cognitive Assessments for Patients on Alcohol Detoxification, in Bridge House Detoxification Unit

**DOI:** 10.1192/bjo.2025.10587

**Published:** 2025-06-20

**Authors:** Sylvia Fatunla

**Affiliations:** Kent and Medway NHS and Social Care Partnership Trust, Kent, United Kingdom

## Abstract

**Aims:** This audit serves to establish the practice in recent time, if patients admitted for alcohol detoxification, were examined physically and offered a cognitive assessment prior to discharge according to NICE guidelines and trust policy, as this will help to reduce missed opportunities for clients to receive effective interventions for co-morbidities.

If not, for necessary changes to be implemented in practice and a re-audit carried out.

**Methods:** The audit was conducted as a retrospective study. We aimed to analyse the records of all patients with alcohol dependence, discharged from Bridge House detoxification unit between July 2024 and October 2024.

National Health Service [NHS] number of all discharged patients between the time period was obtained from our Electronic Discharge Notification [EDN] system and used to access the Rio records, to check if patient was admitted for alcohol detoxification, if physical health examination was done following admission and if cognitive assessment was conducted prior to discharge.

An audit tool was used as reference for data collection, and data was analysed using Excel. In total 59 cases were analysed in this audit.

**Results:** The results showed that more men than women were managed in the inpatient detox unit within the time period, where 33.9% were female, with majority of service clients within working age range.

Physical health check and examination was done for almost all patients (98%). No clear reason stated why this was not done or documented for 1 patient. Cognitive assessment was done for only a few of the clients (7%) admitted to the unit. About 30% had cognitive assessment as part of management plan on initial admission review, but this was not actioned prior to discharge. 1 of the patients declined cognitive assessment when offered.

On average, patients spent about 14 days in the unit for alcohol detoxification.

**Conclusion:** Good practice was upheld by carrying out physical examinations on admission for almost all patients.

Cognitive assessment was not conducted for the majority of the patients prior to discharge and although this was planned on admission for some, the plan was not followed up or actioned prior to discharge. Local best practice is to conduct cognitive assessment as soon as patient is no longer under the influence of alcohol or high doses of benzodiazepines.

